# Novel technique and outcomes of umbilical reconstruction during cytoreductive surgery; a multi-centre study

**DOI:** 10.1007/s10151-024-03095-y

**Published:** 2025-01-21

**Authors:** E. Cheng, P. F. Yang, S. Khor, J. Mui, M. Sarofim, R. Wijayawardana, N. Ansari, C. E. Koh, D. L. Morris, N. Ahmadi

**Affiliations:** 1 Peritonectomy and Liver Cancer Unit, Department of Surgery, St George Hospital, Kogarah, NSW Australia; 2 St George & Sutherland Clinical Campuses, School of Clinical Medicine, University of New South Wales, Sydney, NSW Australia; 3 Department of Colorectal Surgery, Royal Prince Alfred Hospital, Camperdown, NSW Australia; 4 School of Clinical Medicine, South West Sydney Clinical Campuses, University of New South Wales, Sydney, NSW Australia; 5 Faculty of Medicine and Health, Central Clinical School, The University of Sydney, Sydney, NSW Australia; 6 Surgical Outcomes Research Centre (SOuRCe), Royal Prince Alfred Hospital, NSW Sydney, Australia

**Keywords:** Umbilicus, Umbilical reconstruction, Abdominal aesthetics, Cytoreductive surgery

## Abstract

**Background:**

The goal of cytoreductive surgery for peritoneal malignancy is to remove all macroscopic disease, which occasionally requires the excision of the umbilicus. While the absence of the umbilicus can be aesthetically undesirable for patients, umbilical reconstruction is rarely performed due to the perceived complexity and increased risk of wound infections (Sakata et al. in *Colorectal Dis* 23:1153–1157, 2021). This study aims to evaluate the outcomes, cosmetic results and patient satisfaction of umbilical reconstruction during cytoreductive surgery.

**Methods:**

Consecutive patients from a prospectively maintained database who underwent cytoreductive surgery with umbilical excision and reconstruction were evaluated. Our technique for umbilical reconstruction involved recreating the subcutaneous fat space and fashioning umbilical skin flaps that anchor to the anterior fascia. Outcomes assessed included post-operative infection rate, wound dehiscence, seroma formation, wound appearance and patient satisfaction.

**Results:**

Umbilical reconstruction was performed on 50 patients, with 12 (24%) experiencing wound-related complications. Of these, eight patients (16%) had superficial wound infections, while one patient (2%) developed a deep wound infection; three patients (6%) required local wound drainage, though none needed surgical revision. There were no reports of wound seromas, skin necrosis, wound widening nor umbilical stenosis. All patients reported satisfaction with the outcome of their reconstruction.

**Conclusions:**

Our novel technique for umbilical reconstruction during cytoreductive surgery did not negatively impact wound healing outcomes. Recreating the umbilicus improved cosmetic results and patient satisfaction, enhancing body image for those undergoing major abdominal surgery. This approach should be considered for patients undergoing major laparotomies that necessitates umbilical excision.

**Supplementary Information:**

The online version contains supplementary material available at 10.1007/s10151-024-03095-y.

## Introduction

Cytoreductive surgery (CRS) is the mainstay treatment for removal of macroscopic disease in peritoneal malignancies. Complete cytoreduction, achieved through a combination of extensive disease excision and delivery of heated intraperitoneal chemotherapy (HIPEC), has been shown to improve survival in peritoneal surface malignancies [2–4]. To facilitate the removal of all disease, cytoreductive surgery traditionally entails a midline laparotomy and careful resection of involved organs and involved peritoneal surfaces [5]. The umbilicus is hypothesised as a frequent haven for tumour deposits. Sakata et al. found 30% of patients undergoing CRS had disease involving the umbilicus and thus advocated for routine umbilical excision [1]. The infiltration of tumour at the umbilicus can originate from previous diagnostic or staging laparoscopies with port sites acting as a conduit of disease spread, or alternatively, the result of direct spread of disease [6]. Therefore, the risk of incomplete cytoreduction remains a concern if umbilical excision is not performed. Routine excision of the umbilicus is performed in some centres as standard practice for patients with peritoneal surface malignancies.

Crucially, there are body image and cosmetic implications for patients requiring umbilical excision. Patients often perceive the absence of the umbilicus as aesthetically displeasing and may experience significant psychological distress as a result. The umbilicus serves as a noticeable and essential landmark on the abdomen and therefore affects one’s overall appearance and body confidence [7, 8]. Plastic surgeons have recognised both the importance of umbilical appearance and necessity of reconstruction when excised, and therefore have published numerous reconstructive techniques [7–10]. However, a suitable method specifically applicable in the context of CRS and peritoneal malignancy has not yet been described. The aim of this study is to describe a novel technique of umbilical reconstruction during CRS and evaluate post-operative wound complications and patient satisfaction.

## Method

A review of prospectively collected data was conducted on consecutive patients undergoing CRS and HIPEC requiring concurrent umbilical excision between January 2021 and December 2023, at the two largest peritoneal malignancy units in Australia. Written consent was obtained from all patients, and ethics approval was granted by the Human Research Ethics Committee of both Local Health Districts. Patient data collected included age, body mass index (BMI), pre-operative skin height (measured on pre-operative CT scans from skin to fascia at the iliac crest), smoking status, diabetes and intraoperative peritoneal cancer index (PCI) score.

### Surgical technique

**Fig. 1:**
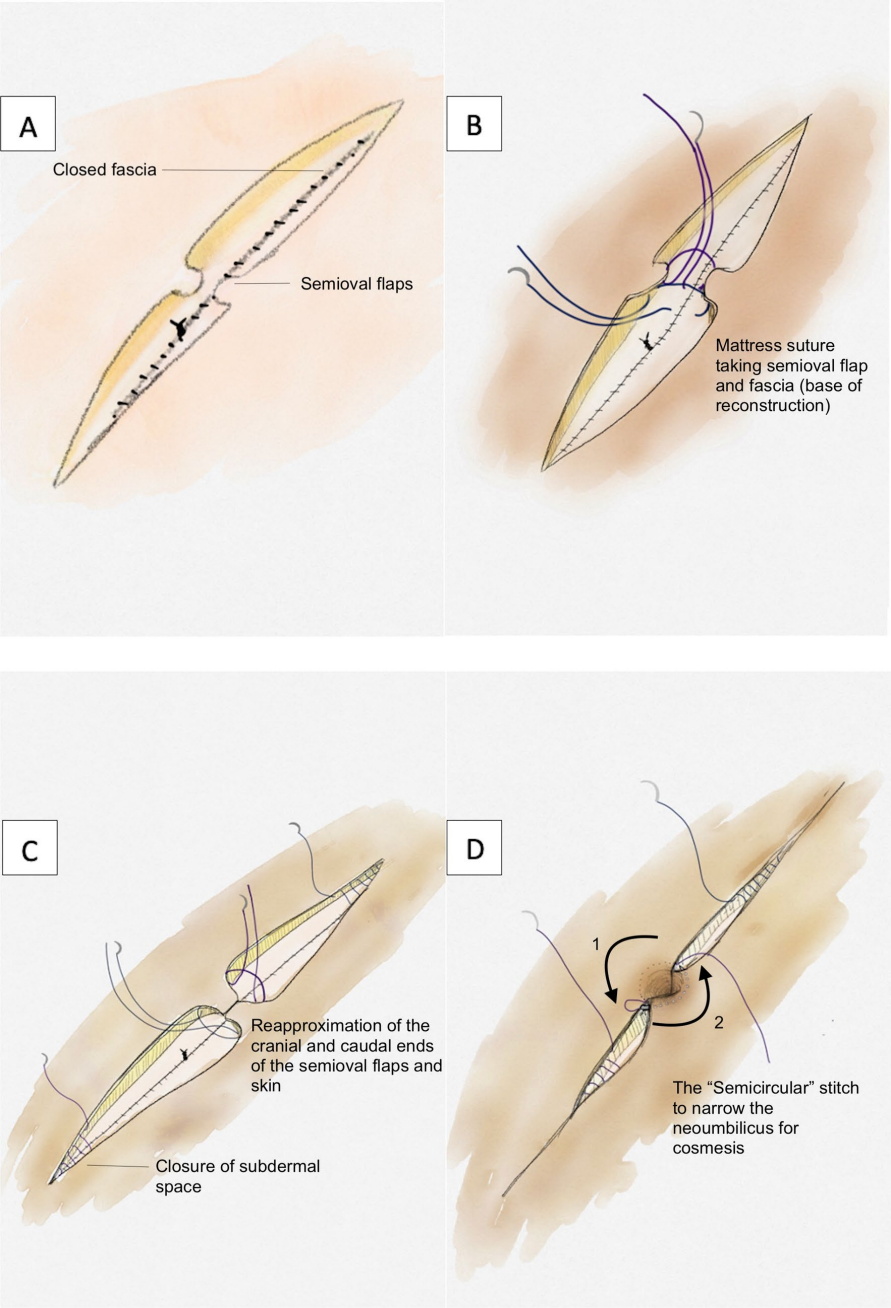
Illustrative representation of umbilical reconstruction after umbilical excision as part of cytoreductive surgery

The same reconstruction technique was performed for all patients. We utilise a standardised laparotomy approach marking the xiphisternum, pubic bone and midline. An elliptical incision is made at the midline to incorporate the umbilicus with the creation of semioval skin flaps at the level of the umbilicus for later reconstruction (Fig. 1A). Entry into the abdomen is made with a combination of diathermy and sharp dissection, and an elongated elliptical wedge of fascia is taken to include the umbilicus. This allows for spread of tension at the time of closure of both fascia and skin. CRS is then performed. Prior to HIPEC, a 2 cm wide subcutaneous dissection is performed at the fascia to expose the anterior sheath and allow for the fascia to be submerged into the HIPEC fluid.

Closure of the fascia is performed using a continuous 1 PDS® (polydioxanone) suture in a standardised manner ensuring that the surgical knots are not placed at the level of the umbilical reconstruction. In patients with a large amount of subcutaneous fat, the fat is removed at the semioval flaps. Two 1 Vicryl® (polyglactin 910) mattress sutures are placed at the cranial and caudal aspect of the vertices of the semioval flaps as well as the fascia and tied down to anchor what will be the base of the umbilical reconstruction (Fig. 1B). Further 1 Vicryl sutures are placed at the cranial and caudal base of the semioval flap on either side and the skin is re-approximated, thus creating a conical shaped umbilical reconstruction (Fig. 1C). A 1 Vicryl suture on a 48 mm needle is then passed parallel to the skin plane in the subcutaneous fat from the cranial apposition suture and removed at the caudal end, ensuring a semicircular path. This is repeated on the other side and tied slowly to narrow the aperture of the neo-umbilicus for cosmesis (Fig. 1D).

The remainder of the wound is closed in two layers; continuous Vicryl subdermal stitches then staples or Monocryl® (poliglecaprone 25) sutures in continuous fashion. No further sutures are placed at the neo-umbilicus to allow for drainage. An alginate dressing is fashioned into a ball and placed in the neo-umbilicus to facilitate drainage and bolster the reconstruction.

### Outcomes

The primary outcome measured was the rate and type of wound complication associated with this umbilical reconstruction technique. This included common wound-related complications such as inflammation and dehiscence as well as both superficial and deep wound infections. We additionally assessed for wound stenosis and widening at the umbilicus. Secondary outcomes were post-operative complications and patient satisfaction using a modified version of the Patient and Observer Scar Assessment Scale (POSAS) [11].

Statistical analyses were performed using IBM SPSS software version 24 (IBM corporation, New York, USA). Descriptive statistics included mean, percentage, standard deviation (SD) and range. Multi-variant analysis was performed to compare between patients with and without complications.

## Results

A total of 50 patients with peritoneal surface malignancy underwent umbilical reconstruction during CRS using our novel technique. The mean age was 56.4 years (SD 11.2, range 30–85 years) with a mean BMI of 27.3 kg/m^2^ (SD 8.24). A summary of patient baseline and operative characteristics is provided in Tables 1 and 2. Wound classification was considered “clean” or “clean-contaminated” in 48 (96%) patients, and mesh was used for abdominal wall reconstruction in four (8%) patients.

**Table 1:** Characteristics of patients who had umbilical reconstruction with cytoreductive surgery

Patient characteristics	Number of patients (%)^a,b^
Age, mean (SD), years	56.4 (11.3)
Sex	
Male	13 (26)
Female	37 (74)
Origin of primary cancer	
Appendiceal	27 (53)
Colorectal	13 (23)
Other (e.g. ovarian, mesothelioma)	10 (23)
ASA score	
1	0
2	3 (6)
3	42 (84)
4	5 (10)
BMI, mean (SD), kg/m^2^	27.3 (8.32)
Pre-operative skin height, mean (SD), mm	23.0 (10.3)
Smoking status	
Current smoker	2 (4)
Ex-smoker	2 (4)
Non-smoker	46 (92)
Diabetes, *n* (%)	
Yes	6 (12)
No	42 (84)

^a^Data are presented as no. of patients (%) unless otherwise stated. Percentages may not total 100 because of rounding

^b^*SD* standard deviation; *ASA* American Society of Anesthesiology; *BMI* body mass index

**Table 2:** Operative characteristics of patients who underwent umbilical reconstruction with their cytoreductive surgery

Operative characteristics	Number of patients (%)^a,b^
PCI score, median (interquartile range)	14 (6–24)
CC score	
0	45 (90)
1	3 (6)
2	2 (4)
Wound classification	
Clean	8 (16)
Clean-contaminated	42 (84)
HIPEC	
None	2 (7)
Mitomycin	41 (82)
Cisplatin	2 (4)
Oxaliplatin	3 (6)
Mitomycin + cisplatin	2 (4)
Use of mesh	4 (8)
Operative time in min, mean (SD)	626 (188)

^a^Data are presented as no. of patients (%) unless otherwise stated. Percentages may not total 100 because of rounding

^b^*PCI* peritoneal cancer index; *CC* completeness of cytoreduction

A total of 12 (24%) of patients experienced wound-related complications (Table 3); 4 showed signs of wound inflammation, 2 of whom subsequently developed superficial surgical site infections requiring antibiotic treatment. The other two patients did not require any treatment. In total, eight patients experienced superficial surgical site infections requiring antibiotic treatment; two of these patients required bedside drainage of the wounds resulting in minor wound dehiscence. One patient experienced a deep surgical site infection requiring antibiotics and washout of their wound performed at the bedside. All complications observed were classified as either grade I or grade II according to the Clavien–Dindo classification scale. None of the patients experience wound seroma, skin necrosis, widening of the wound or umbilical stenosis. Additionally, no patients required radiological interventions for their wound, nor did any require a return to the operating theatres for wound revision procedures. The median hospital length of stay for all patients was 19 days (range 5–47 days).

**Table 3:** Perioperative characteristics and complications in patients with wound complications

Patient no.	Age (years)	Sex	BMI (kg/m^2^)	Skin height (cm)	PCI	Smoking status	Complication
1	62	M	31.2	26	8	Never	Deep SSI
2	69	F	29.5	18	10	Never	Superficial SSI + wound dehiscence
3	53	M	34.9	18	5	Never	Superficial SSI + wound dehiscence
4	46	F	28.3	37	16	Never	Superficial SSI
5	50	F	28.7	17	39	Ex-smoker	Inflammation
6	52	F	23.6	23	26	Ex-smoker	Inflammation + superficial SSI
7	30	F	23.6	27	33	Never	Inflammation + superficial SSI
8	75	F	23.3	29	4	Never	Superficial SSI
9	61	M	27.2	38	18	Never	Inflammation
10	46	F	34.3	30	16	Never	Superficial SSI
11	46	M	26.7	27	16	Never	Superficial dehiscence
12	61	F	29.0	8	25	Never	Superficial SSI

*BMI* body mass index; *PCI* peritoneal cancer index; *SSI* surgical site infection

At routine follow-up at 6 weeks post-operatively, all patients expressed satisfaction with their umbilical reconstruction and did not wish to pursue any further revisional surgery for their umbilical appearance.

**Fig. 2:**
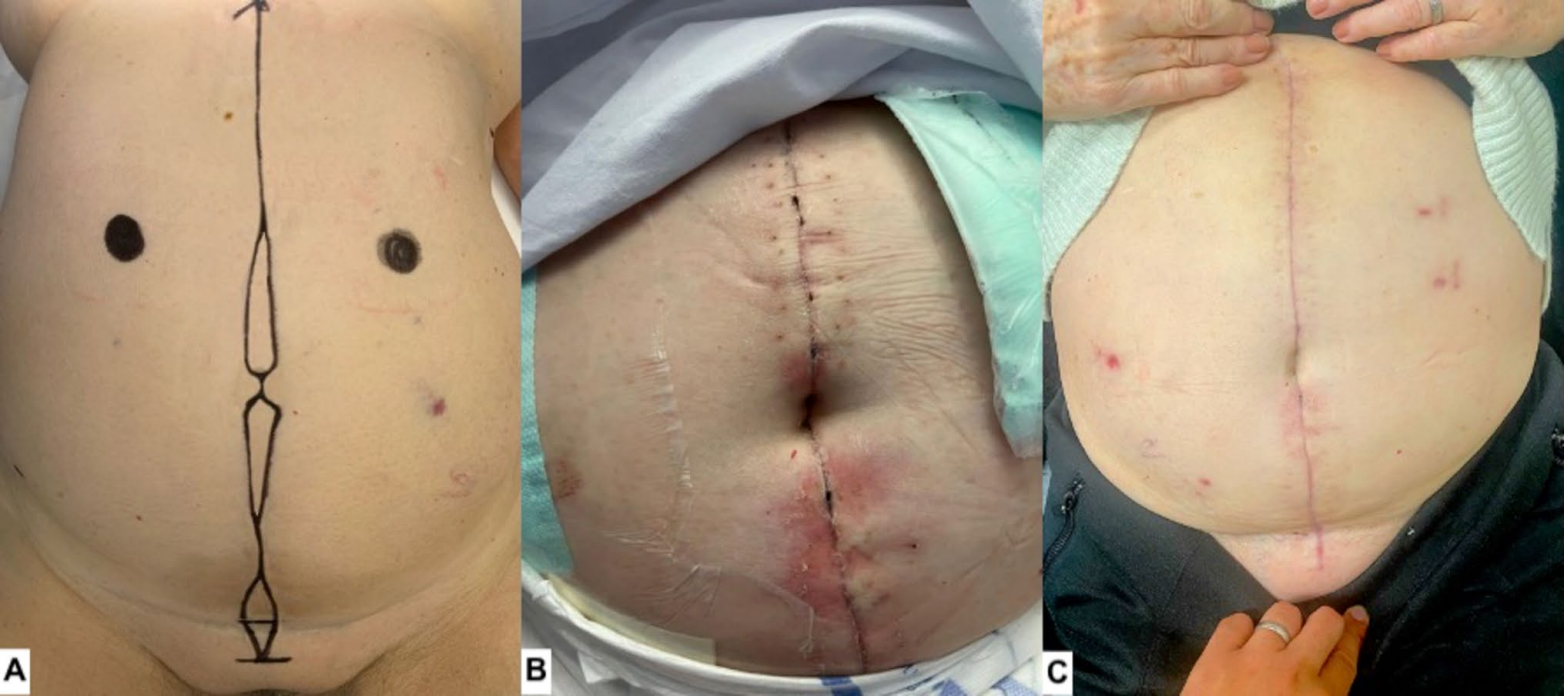
Example of one patient’s abdomen who underwent the described umbilical reconstruction technique. **A** Image taken pre-operatively with incision markings. **B** Image taken 2 weeks post-operatively. **C** Image taken 6 weeks post-operatively

**Fig. 3:**
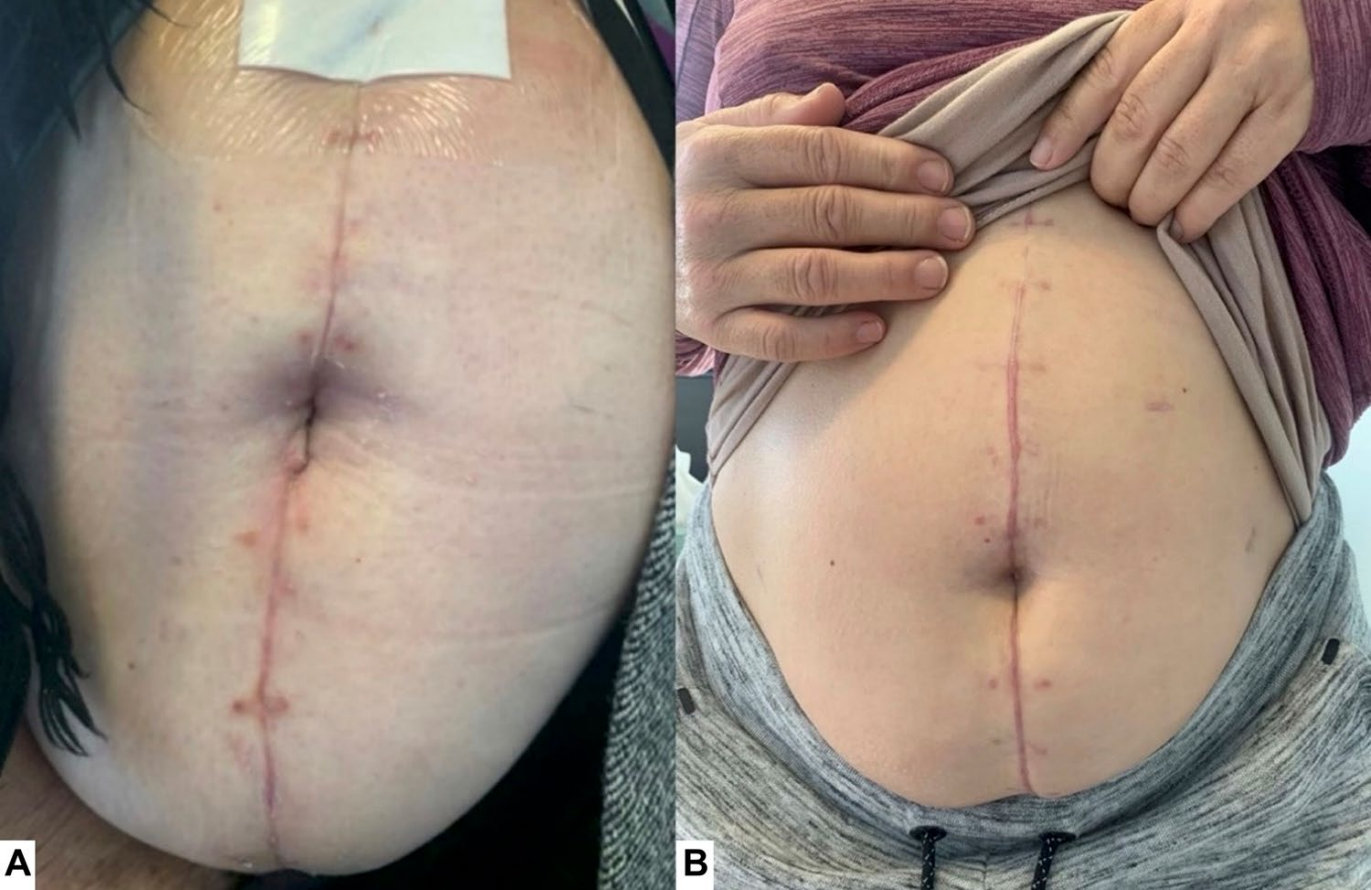
Further examples of umbilical reconstruction outcomes. **A** Image taken 6 weeks post-operatively. **B** Different patient with image taken 8 weeks post-operatively

A total of 72% of patients who completed the POSAS satisfaction survey reported being very satisfied with their umbilical reconstruction, while the remaining 28% were moderately satisfied. Notably, none of the patients expressed dissatisfaction with their scar. The cohort of patients who developed wound-related complications had similarly high patient satisfaction scores as those without complications. Figures 2 and 3 provide a chronological demonstration of patients undergoing the umbilical reconstruction over a 6-week period.

When comparing individuals who experienced wound complications with those who did not, pre-operative factors such as age, pre-operative skin height, BMI and PCI score demonstrated no significant impact on the occurrence of wound complications (Table 4). None of the patients who underwent mesh repairs experienced any wound complications. Additionally, the presence of diabetes did not increase their risk of wound-related complications in our study population.

**Table 4:** Comparison of pre-operative factors for patients with and without wound complications

Variable	Wound complication	No wound complications	p-Value
Age, mean in years	54.3	57.1	0.45
BMI, mean	28.3	26.9	0.61
Skin height, mean in cm	24.9	22.4	0.46
PCI score, median	11.0	16.0	0.19
Wound clean-contaminated	33	9	0.33

## Discussion

Although numerous techniques for umbilical reconstruction have been described in the literature, this series of 50 patients is the first to present a simple and reproducible method of umbilical reconstruction during CRS where excision of the umbilicus is of oncological importance [7, 8]. Our technique is novel due to its ease, applicability and ability to be performed concurrently with CRS. Wound complications were observed in 24% of patients, the majority of whom experienced superficial surgical site infections. The complication rate is consistent with the reported 17–46% wound complication rate in the CRS literature, suggesting our technique does not carry an increased risk of wound complications [12–14]. No patient demographic variables were significantly associated with increased risk of wound complication.

Our data demonstrate satisfactory aesthetic results and high patient satisfaction scores while maintaining an acceptable level of wound complications. Understandably, there may be hesitancy in performing an immediate reconstructive procedure in settings with wounds are at high risk for infection, such as those with faeculant or biliary contamination [15]. However, with early recognition and appropriate antimicrobial treatment of infections, we have demonstrated no significant change in the final cosmetic result. Clinicians should also demonstrate heightened vigilance and awareness when evaluating surgical wounds, especially in cases where reconstruction has taken place.

In contrast to cosmetic surgery, open abdominal surgery for peritoneal malignancy places less (or no) emphasis on body image and cosmesis. Optimal oncological resection is the most important for outcome for surgeons, however, from a patient perspective, improved cosmesis and body image have been shown to be advantageous in improving quality of life, self-esteem and functional outcomes [16]. This is well established in breast cancer surgery, with breast reconstruction and onco-plastics becoming a sub-speciality field of its own [17]. Similarly, one of the many benefits of laparoscopic surgery is fewer scars leading to better cosmesis and improved body image [16, 18]. Unfortunately, considerations of body image are often overlooked in the context of radical cancer surgery for peritoneal malignancy: CRS significantly distorts normal surface anatomy with a midline scar, multiple drain sites and potentially a stoma. However, stomas are often reversed, and scars fade to become less prominent, leaving the lack of the umbilicus a distinct remaining anomaly. The umbilicus serves as a significant landmark and contributes to the natural appearance of the abdomen; therefore, its preservation is important to one’s body image [8].

Complete reconstruction of the umbilicus or “neo-umbilicoplasty” by general surgeons differs from the usual technique of transposing the umbilicus performed after detachment during umbilical hernia operations [7]. With neo-umbilicoplasty, the precise position, symmetry and recreation of a natural-looking umbilicus are essential. This requires careful attention to detail regarding the shape, depth and proportion of the umbilicus. Joseph et al. described the “perfect umbilicus” as a vertically oriented, oval-shaped umbilicus with slight superior hooding [8]. Our described technique incorporates various elements from different techniques reported in the literature. We utilise the vertical ellipse incision and attachment of the umbilicus to the rectus as described by Bruekers et al. and Mazzocchi et al. [19, 20]. Periumbilical defatting was described by several authors and is also a fundamental component in our approach [7, 10]. Admittedly, there are a vast number of reconstructive techniques developed by plastic surgeons who perform abdominoplasties: several involves complex flaps, staged reconstruction and the use of autologous grafts to recreate the natural umbilicus shape and appearance [7]. Our technique is specifically tailored for patients undergoing maximally invasive surgery, acknowledging that achieving a perfect abdominal appearance may not be possible due to the radical nature of surgery. Thus, our aim is to offer a semblance of normalcy in an otherwise battle-scarred abdomen. One advantage of our technique is its ability to be performed concurrently with the index operation and requiring minimal additional time to perform in comparison with the entire operation. Its simplicity also allows for an easy learning curve, and the necessary concepts are readily applicable to general surgeons without requiring plastic surgical input.

This study strength includes being performed in the two highest-volume peritoneal malignancy units in Australia. We of course acknowledge several limitations including its retrospective design. The sample size is small; however, it is important to consider this in the context of the relatively rare incidence of peritoneal malignancy. This study could be strengthened by the inclusion of a direct comparison group of patients who underwent umbilical excision without subsequent reconstruction. Future prospective and randomised studies will be useful to confirm the demonstrated safety and benefits of this initial pilot study. This technique has the potential to extend further than just CRS and is transferable to other midline abdominal incisions requiring umbilical excision such as revisional ventral hernia surgery, endometriosis involving the radical excision of the umbilicus and primary tumours originating near the umbilicus.

## Conclusions

Umbilical reconstruction in patients undergoing CRS is safe and technically feasible, without significant compromise to wound healing outcomes. A meticulously crafted umbilicus not only reinstates the semblance of a visually normal abdomen, but also enhances patients to experience improved self-esteem and body confidence. Our novel technique can be employed by all surgeons to improve cosmesis, patient satisfaction and long-term body image for patients undergoing major abdominal surgery where umbilical excision is required.

## Supplementary Information

Below is the link to the electronic supplementary material.

Supplementary file1 (MP4 87315 KB)

## Data Availability

No datasets were generated or analysed during the current study.
